# Effects of Ambient Temperature on Sleep and Cardiovascular Regulation in Mice: The Role of Hypocretin/Orexin Neurons

**DOI:** 10.1371/journal.pone.0047032

**Published:** 2012-10-08

**Authors:** Viviana Lo Martire, Alessandro Silvani, Stefano Bastianini, Chiara Berteotti, Giovanna Zoccoli

**Affiliations:** Department of Human and General Physiology, Alma Mater Studiorum – University of Bologna, Bologna, Italy; University of Iowa, United States of America

## Abstract

The central neural pathways underlying the physiological coordination between thermoregulation and the controls of the wake-sleep behavior and cardiovascular function remain insufficiently understood. Growing evidence supports the involvement of hypocretin (orexin) peptides in behavioral, cardiovascular, and thermoregulatory functions. We investigated whether the effects of ambient temperature on wake-sleep behavior and cardiovascular control depend on the hypothalamic neurons that release hypocretin peptides. Orexin-ataxin3 transgenic mice with genetic ablation of hypocretin neurons (n = 11) and wild-type controls (n = 12) were instrumented with electrodes for sleep scoring and a telemetric blood pressure transducer. Simultaneous sleep and blood pressure recordings were performed on freely-behaving mice at ambient temperatures ranging between mild cold (20°C) and the thermoneutral zone (30°C). In both mouse groups, the time spent awake and blood pressure were higher at 20°C than at 30°C. The cold-related increase in blood pressure was significantly smaller in rapid-eye-movement sleep (REMS) than either in non-rapid-eye-movement sleep (NREMS) or wakefulness. Blood pressure was higher in wakefulness than either in NREMS or REMS at both ambient temperatures. This effect was significantly blunted in orexin-ataxin3 mice irrespective of ambient temperature and particularly during REMS. These data demonstrate that hypocretin neurons are not a necessary part of the central pathways that coordinate thermoregulation with wake-sleep behavior and cardiovascular control. Data also support the hypothesis that hypocretin neurons modulate changes in blood pressure between wakefulness and the sleep states. These concepts may have clinical implications in patients with narcolepsy with cataplexy, who lack hypocretin neurons.

## Introduction

The thermoregulatory mechanisms controlling heat production and heat loss are coordinated with the wake-sleep behavior (wakefulness; non-rapid-eye-movement sleep, NREMS; rapid-eye-movement sleep, REMS) and cardiovascular functions. Shifts in ambient temperature (T_a_) toward the thermoneutral zone increase the time spent in NREMS and particularly in REMS [1]–[3]. During REMS, the relationship between hypothalamic temperature and metabolic heat production is weak [4] and shivering and panting are absent [5]. These phenomena likely result from changes in the thermal sensitivity of hypothalamic preoptic neurons [4], [6]. On the other hand, blood pressure increases at cold T_a_ [7], [8], and this may explain at least part of the excess cardiovascular mortality in cold winter months [9], [10]. The cold-related increase in blood pressure is particularly evident in rats and mice, in which it is accompanied by marked increases in heart rate [11]. In rats, the cardiovascular effects of cold exposure are different in different wake-sleep behaviors: at cold T_a_, blood pressure and heart rate rise more in NREMS than in REMS, whereas variability of blood pressure is selectively reduced in REMS [12]. However, this information is still missing in mice, which are the mammalian species of choice for functional cardiovascular and sleep genomics.

**Figure 1:**
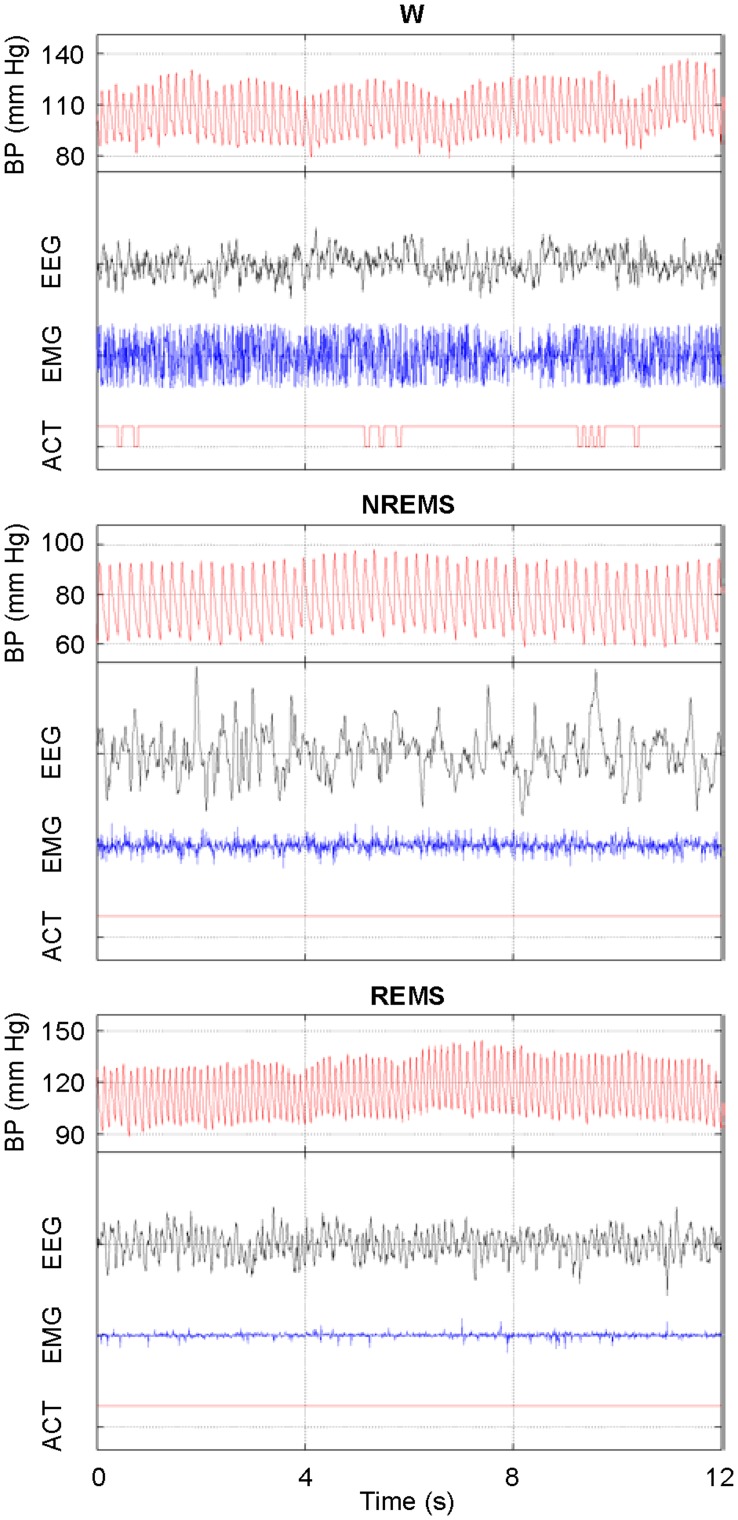
Raw recordings in different wake-sleep states. Representative raw recordings obtained from one orexin-ataxin3 transgenic mouse at ambient temperature of 30°C during wakefulness (W), non-rapid-eye-movement sleep (NREMS) and rapid-eye-movement sleep (REMS). BP, blood pressure; EEG, electroencephalogram; EMG, nuchal muscle electromyogram; ACT, activity. W was scored when the EMG tone was high and the EEG was at low voltage with possible δ (0.5–4 Hz) and θ (6–9 Hz) frequency components. NREMS was scored when the EMG tone was lower than in W and the EEG was at higher voltage with prominent δ frequency components. REMS was scored when the EMG indicated muscle atonia with occasional muscle twitches and the EEG was at low voltage with predominant θ frequency components [25].

The central neural pathways underlying thermoregulation are being increasingly elucidated (cf. [13], [14], for recent reviews). In particular, information on T_a_ is sensed by skin receptors and relayed to the hypothalamus, where it is integrated with the activity of temperature-sensitive neurons in the medial preoptic area to modulate inhibitory projections to the rostral medullary raphe region. The raphe eventually activates the thermoregulatory effectors, such as brown adipose tissue and skin blood vessels, which are involved in defence against cold [13], [14]. However, the central neural pathways that underlie the coordination between thermoregulation, control of wake-sleep behavior, and autonomic cardiovascular control still remain poorly understood [14]. Different lines of evidence suggest that the hypothalamic neurons that release hypocretin (HCRT)/orexin peptides may play an important role in these pathways. HCRT neurons receive projections from the hypothalamic medial preoptic area [15]. Inhibition of the preoptic area activates HCRT neurons [16] and elicits increases in heat production, heart rate, and blood pressure, which are blunted by HCRT-1 receptor blockade [17]. Moreover, blood pressure and heart rate increase following HCRT-1 microinjection in the rostral medullary raphe [18]. On the other hand, the firing rate of HCRT neurons is wake-sleep dependent, decreasing from active wakefulness to quiet wakefulness, NREMS, and REMS, with the exception of occasional bursts in REMS [19]. Moreover, the lack of HCRT neurons in patients with narcolepsy-cataplexy [20] or in mouse models [21] entails substantial consequences on the wake-sleep behavior, which include fragmentation of wakefulness, reduced latency of REMS episodes, and cataplexy. Intracerebroventricular HCRT administration increases blood pressure and renal sympathetic nerve activity in conscious rats [22]. Recent data also indicate that sleep-dependent changes in blood pressure are blunted in mouse models [23] and narcolepsy-cataplexy patients [24] lacking HCRT neurons, suggesting that HCRT neurons play a role in sleep-related cardiovascular control.

**Figure 2:**
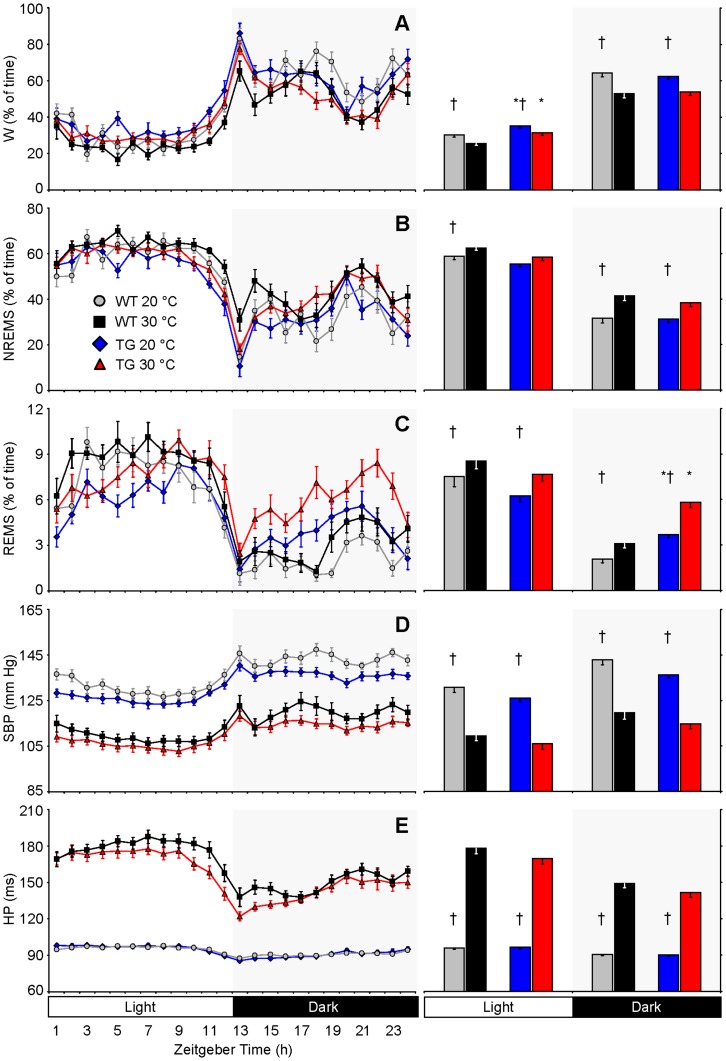
Daily profiles of wake-sleep behavior and cardiovascular variables. Daily profiles (left) and 12-h average values for the light and dark periods (right) of the percentage of recording time spent in wakefulness (W, panel A), non-rapid-eye-movement sleep (NREMS, panel B) and rapid-eye-movement sleep (REMS, panel C), of systolic blood pressure (SBP, panel D), and of heart period (HP, panel E). Zeitgeber time is expressed in hours from the onset of the light period. Data are mean values ± SEM in orexin-ataxin3 transgenic mice (TG, n = 11) and wild-type controls (WT, n = 12) recorded at ambient temperatures (T_a_) of 20°C and 30°C. Statistical analysis was performed on average values for the light and dark periods. * and †, P<0.05, vs. WT and vs. 30°C T_a_, respectively (t-test). Detailed ANOVA results are reported in Table S1.

To summarize, thermoregulation is known to be highly coordinated with sleep [1]–[3] and cardiovascular control [7], [8], [11], [14], but the underlying central neural mechanisms are still poorly understood. Different lines of evidence are consistent with roles of HCRT neurons in thermoregulatory pathways [16]–[18], sleep [19]–[21], and cardiovascular control [22]–[24]. In this study, we investigated the role of HCRT neurons in coordinating thermoregulation with sleep and cardiovascular control. To this aim, we simultaneously recorded wake-sleep behavior, systolic blood pressure (SBP), and heart period (HP) in hybrid transgenic mice with genetic ablation of HCRT neurons [21] at different values of T_a_. We studied hybrid mice with mixed genetic background to provide a first step of approximation to genetic variability in patients with narcolepsy with cataplexy. We tested the hypothesis that the effects of T_a_ on sleep and cardiovascular control were reduced or absent in these transgenic mice lacking HCRT neurons compared with wild-type mice that normally express HCRT neurons.

## Methods

The experiments were performed at the Laboratory of Physiological Regulation in Sleeping Mice (PRISM), Dept. of Human and General Physiology, Alma Mater Studiorum - University of Bologna, and the Centre for Applied Biomedical Research (CRBA), S. Orsola University Hospital, Bologna, Italy.

**Table 1:** Analysis of wake-sleep structure.

			WT		TG		
State	Measure	ANOVA	20°C	30°C	20°C		30°C
W	eps/24 h	b	137±8	141±4	214±16*	213±14*	
	D (s)	a,b	302±24^†^	234±10	202±15*	175±12*	
	ACT (au)	b	14±1	12±1	9±1*	10±1	
NREMS	eps/24 h	a*x*b	448±25^†^	485±24	481±20	452±18	
	D (s)	a, a*x*b	91±7	95±6	78±3^†^	93±4	
REMS	eps/24 h		67±5	71±6	74±7	79±6	
	D (s)		61±2^†^	72±3	60±4^†^	75±3	
	latency (s)	a,b	504±43^†^	739±67	281±26*†	392±51*	

W, wakefulness; NREMS, non-rapid-eye-movement sleep; REMS, rapid-eye-movement sleep; eps/24 h, number of episodes in 24 hours; D, episode duration; ACT, motor activity (arbitrary units, au); latency, time elapsed between onset of REMS episodes and termination of previous W episodes. Data are mean ± SEM in orexin-ataxin3 transgenic mice (TG, n = 11) and wild-type controls (WT, n = 12) recorded at ambient temperatures (T_a_) of 20°C and 30°C. a, b, and a*x*b, *P*<0.05 for main effects of T_a_ and mouse group and for interaction effect, respectively (ANOVA).

* and ^†^,*P*<0.05 vs. WT and vs. 30°C T_a_, respectively (*t*-test).

### Ethical Approval

The study protocol was approved by the Bologna University ethics committee on animal experimentation and the Italian Ministry of Health and complied with the National Institutes of Health guide for the care and use of laboratory animals.

### HCRT-deficient Mouse Models of Narcolepsy

Experiments were performed on male age-matched (14.4±0.4 wks) orexin-ataxin3 transgenic mice (TG [21], n = 11) and wild-type control littermates (WT, n = 12). TG were hemizygous for a transgenic construct coding for the human neurotoxin Ataxin-3 under the control of the HCRT gene promoter. As a result of transgene expression, HCRT neurons are selectively and progressively destroyed in TG after birth [21]. The mice under study did not differ significantly in terms of body weight (29.2±0.7 g and 28.6±0.8 g at surgery in TG and WT, respectively, *P* = 0.58, *t*-test). Mice were bred and housed under a 12:12-h light-dark cycle with T_a_ set at 25°C and free access to water and food (4RF21 diet, Mucedola, Settimo Milanese, Italy). Founder orexin-ataxin3 transgenic mice were generously provided by Prof. E. Mignot (Stanford University, USA) and colony expansion has been described in detail elsewhere (cf. Data Supplement to Ref. [23]). To obtain TG and WT littermates, male mice hemizygous for the orexin-ataxin3 transgene and fully congenic to C57Bl/6J were crossed with female (C57Bl/6J × DBA/2J)F1 hybrid mice, yielding a progeny with 75% C57Bl/6J and 25% DBA/2J genetic background. Mouse genotype was determined by PCR on DNA extracted from tail biopsies. The lack of measurable brain levels of HCRT-1 peptide in TG was verified in a subset of mice (n = 4 TG and n = 5 WT) by means of fluorescent immunoassay (FEK 003-30, Phoenix Pharm. Inc., Burlingame, CA, USA) with techniques previously described in detail (cf. Data Supplement to Ref. [23]). In particular, all samples were processed simultaneously on the same immunoassay plate with a manufacturer-determined detection threshold of 20.3 pg HCRT1/mL. The measured intra-assay coefficient of variation was 2.3%. As expected, brain HCRT1 was below detection threshold in each TG mouse, whereas it was above detection threshold in each WT mouse (116±21 fmol HCRT1/mg total protein, mean ± SEM, n = 5).

**Figure 3:**
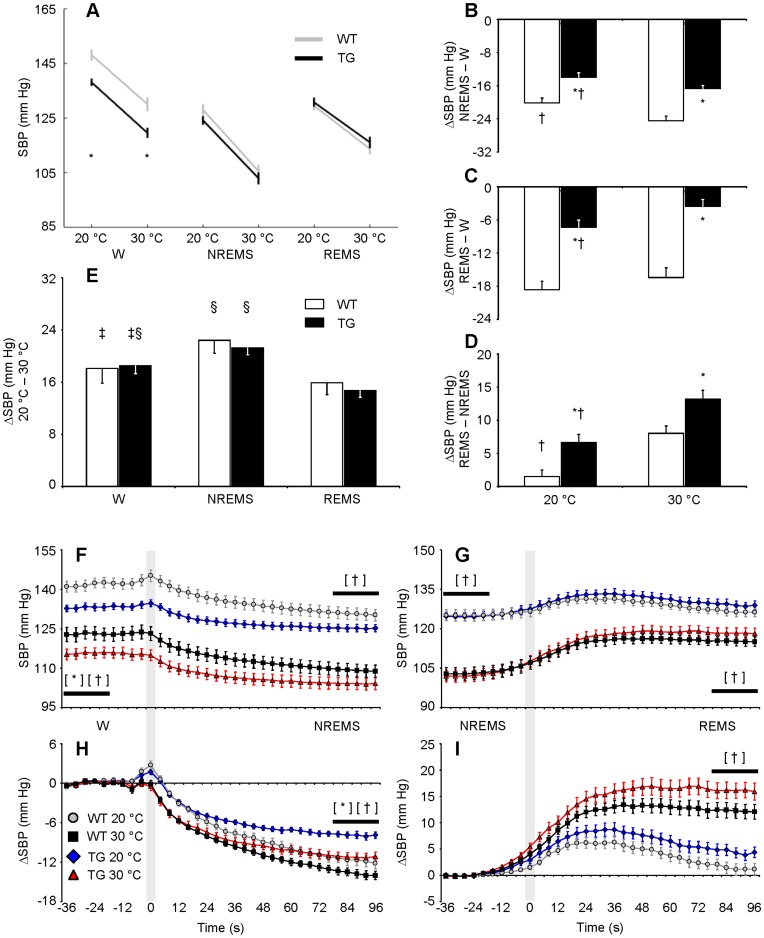
Effects of sleep and ambient temperature on systolic blood pressure. Panel A shows SBP as a function of the wake-sleep state and T_a_. Panels B–D show SBP differences (ΔSBP) between pairs of wake-sleep states. Panel E shows ΔSBP between 20°C T_a_ and 30°C T_a_ in each state. Panels F and G show SBP during transitions between W and NREMS and between NREMS and REMS, respectively. Statistical analysis was performed on 20-s periods (horizontal bars) before (baseline) and after state transitions. Panels H and I show ΔSBP during state transitions with respect to baseline. Gray shadings at time 0 mark boundaries between states. Data are mean values ± SEM in TG (n = 11) and WT (n = 12). *, †, ‡, and §, *P*<0.05 vs. WT, vs. 30°C T_a_, vs. NREMS, and vs. REMS, respectively (*t*-test). [*], *P*<0.05, TG vs. WT at each T_a_ (*t*-test). [†], *P*<0.05, 20°C T_a_ vs. 30°C T_a_ in each mouse group (*t*-test). Detailed ANOVA results are reported in Table S2. Abbreviations have the same meaning as in Figure 2.

### Experimental Protocol

Mice were instrumented with a fronto-parietal differential electroencephalographic lead, a differential electromyographic lead from nuchal muscles, and a telemetric blood pressure transducer (TA11PAC10, Data Science International, Tilburg, The Netherlands) under general anesthesia (isoflurane 1.8–2.4% in O_2_) and intraoperative analgesic treatment (Carprofen 0.1 mg s.c., Pfizer Italy, Latina). The catheter of the blood pressure transducer was inserted through the femoral artery and its tip was advanced a pre-determined length to reach the abdominal aorta below the renal arteries. The body of the transducer was implanted subcutaneously on the mouse flank. The telemetric system also yielded a motor activity signal based on the shifts of the implanted transducer with respect to the external receiver. The electroencephalographic and electromyographic signals were transmitted via cable and synchronized to the telemetric signals by means of simultaneous analog-to-digital conversion [25]. A rotating swivel (SL2+2C/SB, Plastics One, Roanoke, VA, USA) and a balanced suspensor arm prevented the cable from twisting and sustained its weight, thus allowing unhindered movements to the mice. After surgery, mice were allowed a minimum of 18 days of recovery, the last 7 of which included habituation to the recording cable setup. Thereafter, 6 TG and 6 WT had T_a_ increased from 25°C to 30°C, which is closer to, if not within, the thermoneutral zone for mice [11], [26]. After 24 h of habituation, these mice underwent continuous recordings for 48 h at 30°C T_a_ while undisturbed and freely-moving in their own cages. Immediately afterwards, T_a_ was brought back to 25°C for 24 h, then further decreased to 20°C. After 24 h of habituation, these mice were again recorded for 48 h at 20°C T_a_. Another randomly-chosen subset of 5 TG and 6 WT had recordings performed with T_a_ changes in reverse order (i.e., first at 20°C T_a_, then at 30°C T_a_).

**Figure 4:**
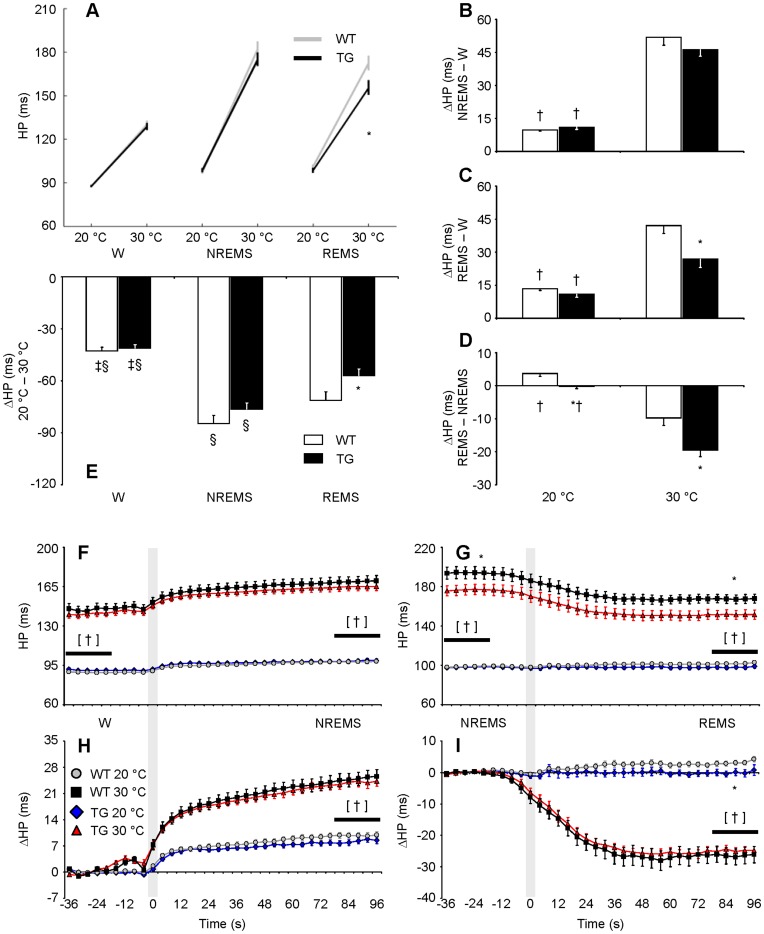
Effects of sleep and ambient temperature on heart period. Panel A shows HP as a function of the wake-sleep state and T_a_. Panels B–D show HP differences (ΔHP) between pairs of wake-sleep states. Panel E shows ΔHP between 20°C T_a_ and 30°C T_a_ in each state. Panels F and G show HP during transitions between W and NREMS and between NREMS and REMS, respectively. Statistical analysis was performed on 20-s periods (horizontal bars) before (baseline) and after state transitions. Panels H and I show ΔHP during state transitions with respect to baseline. Gray shadings at time 0 mark boundaries between states. Data are mean values ± SEM in TG (n = 11) and WT (n = 12). *, †, ‡, and §, *P*<0.05 vs. WT, vs. 30°C T_a_, vs. NREMS, and vs. REMS, respectively (*t*-test). [†], *P*<0.05 vs. 30°C T_a_ in each mouse group (*t*-test). Detailed ANOVA results are reported in Table S2. Abbreviations have the same meaning as in Figure 2.

### Data Analysis

Scoring of wake-sleep states (wakefulness, NREMS, and REMS) was performed visually on the basis of raw electroencephalographic (EEG) and nuchal muscle electromyographic (EMG) recordings with 4-s resolution [25] (Figure 1). For the purpose of wake-sleep structure analysis, minimal duration of wakefulness and sleep episodes was set at 12 s [23]. REMS episodes at sleep onset were scored as cataplexy-like states when they were preceded by episodes of wakefulness longer than 40 s in agreement with consensus criteria of the International Working Group on Rodent Models of Narcolepsy [27]. Beat-to-beat values of SBP and HP were computed as the maximum blood pressure values at each systolic upstroke and as the time intervals between the onset of successive systolic upstrokes of blood pressure, respectively [25].

The spontaneous fluctuations of SBP and HP were analyzed on artefact-free wake-sleep episodes of duration ≥60 s as previously described [28]. In particular, the total variability of HP and SBP was quantified by the standard deviation of the respective values within each wake-sleep episode (indexes SDHP and SDSBP). The cardiac baroreflex sensitivity (BRS) estimating cardiac baroreflex gain was computed with the sequence technique [28], [29]. The contributions of the baroreflex and central autonomic commands to cardiac control were estimated by computing HP vs. SBP cross-correlation functions (CCF) and coherent averaging of spontaneous SBP surges [28]. All data analysis procedures were performed with custom software written in MatLab (Mathworks, Natick, MA, USA).

**Figure 5:**
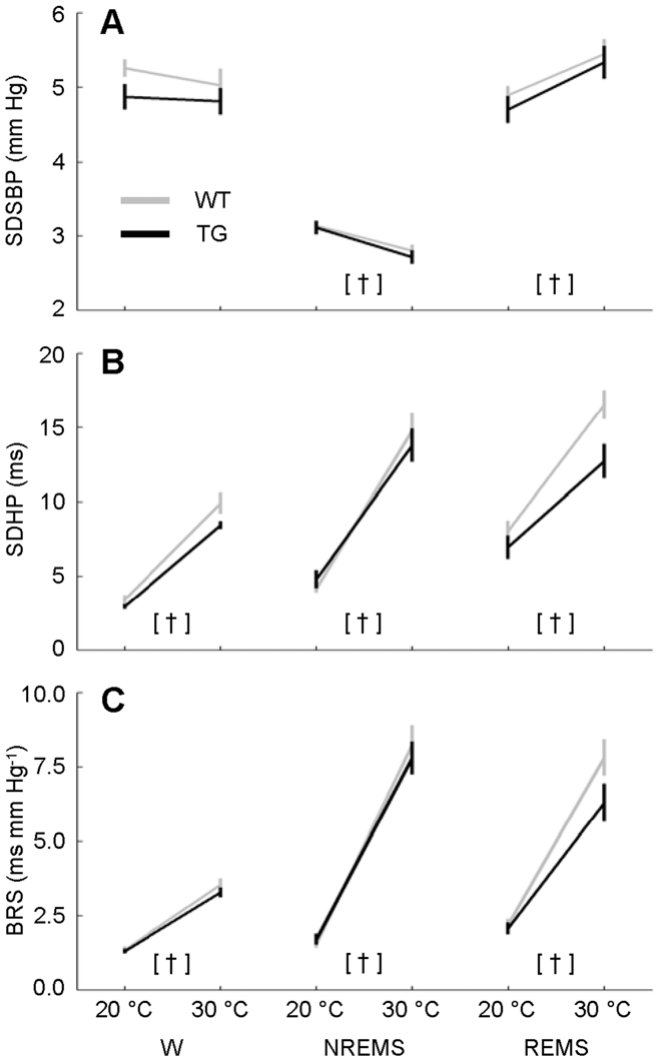
Total variability of systolic blood pressure and heart period and spontaneous baroreflex sensitivity at different ambient temperatures. Total variability of systolic blood pressure (SDSBP, panel A) and heart period (SDHP, panel B) and spontaneous cardiac baroreflex sensitivity (BRS, panel C) as a function of the wake-sleep state and T_a_. Data are mean values ± SEM in TG (n = 11) and WT (n = 12). [†], *P*<0.05, 20°C T_a_ vs. 30°C T_a_ in each mouse group (*t*-test). Detailed ANOVA results are reported in Table S3. Abbreviations have the same meaning as in Figure 2.

**Figure 6:**
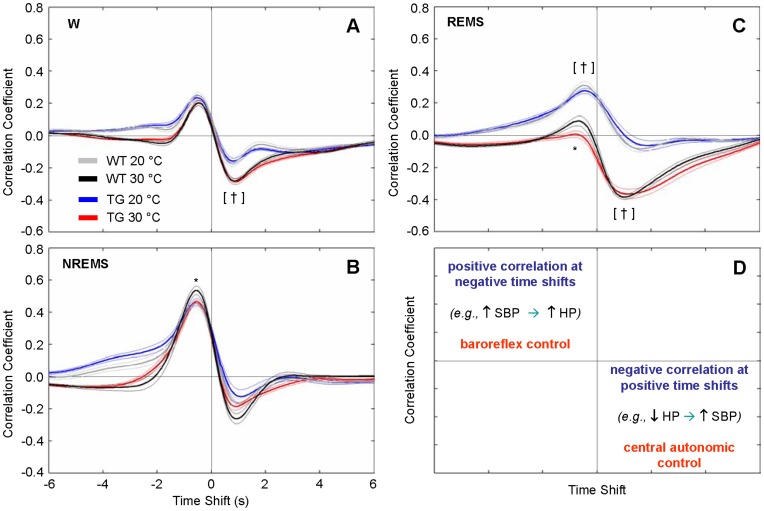
Cross-correlation functions between spontaneous fluctuations of heart period and systolic blood pressure at different ambient temperatures. Panels A–C show cross-correlation functions (CCF) between low-frequency (<0.8 Hz) spontaneous fluctuations of HP and SBP as a function of the wake-sleep state and T_a_. Data are mean values ± SEM in TG (n = 11) and WT (n = 12). Panel D summarizes the interpretation of CCF results [28], [31]. Negative time shifts indicate that changes in HP follow those in SBP. A positive CCF peak at negative time shifts indicating, e.g., cardiac slowing after an increase in blood pressure, is consistent with baroreflex buffering of SBP changes elicited by vascular resistance fluctuations. A negative CCF trough at positive time shifts indicating, e.g., cardiac acceleration before an increase in blood pressure, is consistent with central autonomic commands acting on the heart. Accordingly, statistical analysis (panels A–C) was performed on the correlation coefficients at the positive peak and at the negative trough values of the CCF. *, *P*<0.05, TG vs. WT (same T_a_, *t*-test). [†], *P*<0.05, 20°C vs. 30°C in each mouse group (*t*-test). Detailed ANOVA results are reported in Table S4. Abbreviations have the same meaning as in Figure 2.

**Figure 7:**
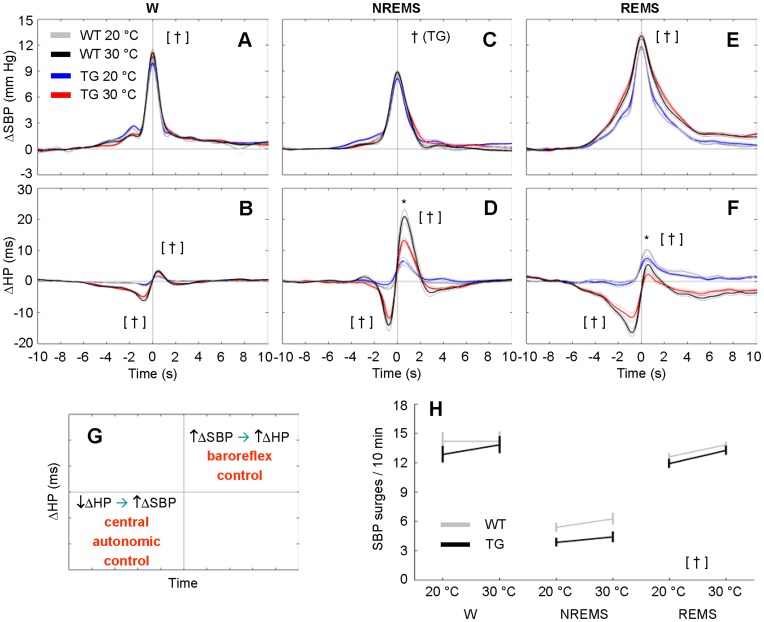
Coherent averaging of spontaneous surges of blood pressure at different ambient temperatures. The panels show time series of systolic blood pressure (ΔSBP, panels A, C, E) and heart period (ΔHP, panels B, D, F) during spontaneous SBP surges (detection threshold = 5 mm Hg) as a function of the wake-sleep state and T_a_. The time series were normalized by subtraction of their respective baseline values and synchronized at the peak SBP increase. Panel G summarizes the interpretation of ΔHP during spontaneous SBP surges [28], [31]. The negative ΔHP trough (i.e., cardiac acceleration) before the SBP surge peak is consistent with central autonomic control of the heart. The positive ΔHP peak (i.e., cardiac slowing) after the SBP surge peak is consistent with baroreflex cardiac control. Accordingly, the statistical analysis in panels A–F was performed on the peaks of ΔSBP and ΔHP and on the trough of ΔHP. Panel H shows the frequency of occurrence of spontaneous SBP surges expressed as the number of surges in 10 min of each wake-sleep state. Data in all panels are mean values ± SEM in TG (n = 11) and WT (n = 12). * and †, *P*<0.05, TG vs. WT (same T_a_) and 20°C T_a_ vs. 30°C T_a_ (same group), respectively (*t*-test). The † symbol between brackets applies to each mouse group. Detailed ANOVA results are reported in Table S5. Abbreviations have the same meaning as in Figure 2.

### Statistics

Statistical analysis was performed with SPSS software (SPSS, Chicago, IL, USA) with significance at *P*<0.05. Data were analyzed with mixed-model ANOVA (GLM procedure with Huynh-Feldt correction), factors being T_a_ and the mouse group and including the wake-sleep state or the light-dark period in different analyses. Simple effects of T_a_, the mouse group, or the wake-sleep state were analyzed with Student’s *t*-tests, and false-discovery rate correction for multiple comparisons [30] was applied when the ANOVA highest-order interaction effect was not significant. Data were reported as means ± SEM with n = 11 TG and n = 12 WT.

## Results

### Daily Profiles of Wake-sleep Behavior and Cardiovascular Variables

We found specific effects of the mouse genotype on the daily wake-sleep profiles: TG spent more time in wakefulness during the light period and more time in REMS during the dark period compared with WT, but these differences occurred irrespective of T_a_ (Figure 2; cf. Table S1 for statistical detail). T_a_ significantly altered the daily profiles of wake-sleep behavior in TG as well as in WT. In particular, at 20°C, the time spent awake was higher than at 30°C in both TG and WT, and the time spent in NREMS and REMS was generally lower at 20°C T_a_ than at 30°C T_a_ (the difference in NREMS time in TG during the light period fell short of statistical significance, *P* = 0.07). SBP and HP were substantially higher and lower, respectively, at 20°C than at 30°C in TG as well as in WT.

### Wake-sleep Structure

TG showed a narcoleptic phenotype, with shorter and more frequent episodes of wakefulness and shorter REMS latency compared with WT (Table 1). These differences occurred irrespective of T_a_. Cataplexy-like episodes [27] occurred in 8 out of 11 TG at 20°C and in 6 out of 11 TG at 30°C at rates of 5.1±2.4 and 0.9±0.2 episodes in 24 h, respectively. T_a_ exerted significant effects on the wake-sleep structure of both TG and WT. In particular, the duration and latency of REMS episodes were lower at 20°C than at 30°C in both groups of mice. However, some of the effects of T_a_ showed differences between TG and WT: at 20°C, TG reduced the duration of NREMS episodes, while WT reduced the occurrence rate of NREMS episodes and showed episodes of wakefulness significantly longer than at 30°C.

### Effects of Sleep and Ambient Temperature on Mean Values of Systolic Blood Pressure

TG and WT showed significant differences in the effects of sleep on the mean values of SBP, but not in those of T_a_. In particular, SBP was lower in TG than in WT in wakefulness, but not during sleep (Figure 3A; cf. Table S2 for statistical detail), indicating that the physiologic sleep-dependent reduction of SBP is blunted in TG compared with WT (Figure 3B–C). Accordingly, the analysis of transitions between full-blown wake-sleep episodes indicated that although SBP was lower in TG than in WT during wakefulness, this difference disappeared later on during NREMS (Figure 3F–G). Differences in average SBP values between NREMS and REMS were also greater in TG than in WT at both T_a_ (Figure 3D). This effect fell just short of statistical significance when investigated on between-state transitions (*P* = 0.05 and *P* = 0.08 at 20°C and 30°C, respectively, Figure 3, I). Finally, T_a_ altered the effects of sleep on SBP in TG and WT alike. In particular, the decrease in SBP between wakefulness and NREMS (Figure 3B,H) and the increase in SBP between NREMS and REMS (Figure 3D,I) were smaller at 20°C than at 30°C in both TG and WT. The cold-related increase in SBP did not differ significantly between TG and WT in any wake-sleep state and was the greatest during NREMS in both mouse groups (Figure 3E).

### Effects of Sleep and Ambient Temperature on Mean Values of Heart Period

In TG, T_a_ exerted significant effects on the mean value of HP, and these effects differed quantitatively compared with WT. In particular, in TG, HP was lower than in WT during REMS at 30°C (Figure 4A; cf. Table S2 for statistical detail). This difference originated during NREMS before the transition to REMS (Figure 4G). Moreover, HP in REMS was less responsive to T_a_ in TG than in WT (Figure 4E). However, the cold-related decrease in HP remained the least pronounced in wakefulness and the most pronounced in NREMS in TG as well as in WT. The effects of sleep on the mean value of HP were generally blunted at 20°C compared with 30°C, with quantitative differences between TG and WT (Figure 4C–D). In particular, at 20°C, HP remained almost constant on passing from NREMS to REMS in TG, whereas it increased slightly in WT (Figure 4I).

### Effects of Sleep and Ambient Temperature on Cardiovascular Variability

T_a_ prominently affected indexes of cardiovascular variability and BRS in TG and WT alike. In particular, 20°C T_a_ increased total SBP variability in NREMS, reduced total SBP variability in REMS, and substantially reduced total HP variability and BRS in each wake-sleep state compared with 30°C (Figure 5; cf. Table S3 for statistical detail). The CCF between HP and SBP (Figure 6) provided information on how effectively HP variability could be explained based on the arterial baroreflex and central autonomic commands [31]. In particular (Figure 6D), the positive CCF peak at negative time shifts is consistent with baroreflex buffering of SBP changes elicited by vascular resistance fluctuations, whereas the negative CCF trough at positive time shifts is consistent with central autonomic commands acting on the heart [31]. The CCF analysis revealed that during REMS, the relative contribution of the baroreflex to cardiac control was enhanced, whereas the relative contribution of central commands was reduced at 20°C compared with 30°C in TG and WT alike (Figure 6; cf. Table S4 for statistical detail). The latter effect of T_a_ also occurred, albeit less dramatically, in wakefulness in TG and WT. Significant differences between the 2 groups of mice, consisting of a blunted baroreflex CCF pattern in TG, only emerged at 30°C during NREMS and REMS. Further insight into the cardiovascular effects of T_a_ was offered by the analysis of short-lasting increases (surges) of SBP (Figure 7), which occur spontaneously in mice during each wake-sleep state [28]. The frequency of occurrence of these SBP surges was lower at 20°C than at 30°C in TG and WT alike during REMS only (Figure 7H, cf. Table S5 for statistical detail). The peak SBP increase during the surges was slightly lower at 20°C than at 30°C, although this difference was not significant during NREMS in WT (*P* = 0.15; Figure 7A,C,E). The coherent averaging of the HP fluctuations temporally associated with the SBP surges (Figure 7 B,D,F) confirmed that on average, HP decreases below baseline before the SBP surge peak and/or increases above baseline thereafter [28]. These HP changes inform on the amplitude of the cardiac responses consistent with central autonomic commands and with baroreflex control, respectively [31] (Figure 7G). In TG and WT alike, these responses were blunted at 20°C compared with 30°C in each wake-sleep state, with the exception of the late increase in HP during REMS, which was enhanced at 20°C (Figure 7 B,D,F; cf. Table S5 for statistical detail). Moreover, the late HP peak consistent with baroreflex control was significantly reduced in TG compared with WT during NREMS at 30°C and during REMS at 20°C.

## Discussion

Our study yielded 3 main findings. First, the effects of mild cold T_a_ on the wake-sleep behaviour and cardiovascular control are preserved in TG, which lack HCRT neurons. Second, the decrease in SBP between wakefulness and the sleep states was blunted in TG compared with WT not only at thermoneutral temperature, but even at mild cold T_a_. This is of particular interest in view of our third finding, that T_a_ modified quantitatively and qualitatively the effects of sleep on cardiovascular control in mice of both groups.

Even if our data clearly indicated that the lack of HCRT neurons in TG did not prevent the occurrence of general effects of mild cold T_a_ on the wake-sleep behaviour and cardiovascular control, we found that the effects on NREMS structure (Table 1) and on the control of HP during REMS (Figure 4) showed quantitative modifications in TG compared to WT. This represents evidence that HCRT neurons may modulate the effects of T_a_ on wake-sleep structure and cardiac control, but are not necessary for their occurrence, at least in mice and in physiological conditions of mild cold T_a_. This conclusion based on our novel findings challenges present views based on neuroanatomical and pharmacological evidence, which links HCRT neurons with the central neural pathways of thermoregulation [16]–[18], [32]. Further experiments are needed to extend our conclusion to the long-term effects of T_a_, to the effects of high T_a_, or to severe cold stress.

Our experiments were performed on hybrid TG and WT littermate mice with mixed genetic background (cf. Methods). It is widely recognized that there is no ideal genetic background for mutation experiments [33] because any genetic background can overshadow or exacerbate a specific mutant phenotype by means of epistasis, i.e. the interaction between two or more genes to control a single phenotype [34]. The genetic background of hybrid mice is defined, reproducible, and statistically identical at population level, but differs randomly between individual mice. While this controlled genetic variability may mask a weak phenotype [34], it provides a first step of approximation to human genetic variability, such as that in patients with narcolepsy with cataplexy, whom TG mice were developed to model [21]. Our data indicate that the relatively modest and controlled genetic background variability of hybrid mice made unnecessary the role of HCRT neurons in coordinating thermoregulation with behavioural and cardiovascular control, suggesting that, in this respect, the physiologic significance of HCRT activity is negligible. The specific mixed background of our hybrid TG and WT mice is permissive to metabolic abnormalities (i.e., late-onset obesity) consequent to HCRT neuron loss compared with the pure C57Bl/6J genetic background [21], [35], thus showing another trait of narcoleptic patients [36]. Notably, in order to avoid confounding effects associated with obesity, we started experiments at the age at which obesity just starts to ensue in these mice [21], [35].

The magnitude of the cardiovascular effects of T_a_ that we found in TG and WT (Figures 3 and 4) agrees well with previous work performed on wild-type mice without taking the wake-sleep state into account [11]. Our novel finding is that T_a_ modifies quantitatively and qualitatively the effects of sleep on cardiovascular control in mice, in broad agreement with previous work on rats [12]. We also showed that T_a_ substantially affects not only BRS values (Figure 5), but also HP fluctuations driven by central autonomic commands (Figure 7) in each wake-sleep state in mice. Strikingly, at 30°C, HP variability during REMS was explained almost solely based on these central autonomic commands in TG and WT, in stark contrast with NREMS (Figure 6). This result reconciles data obtained in REMS on mice with those on rats [37] and human subjects [38]. During wakefulness, the reduced effectiveness of central autonomic HP control at 20°C may be because low mean values of HP in wakefulness limit further HP decreases driven by the central commands. This explanation is less plausible during REMS, when the mean HP value was not the lowest among wake-sleep states (Figure 4). Rather, central autonomic commands acting on HP in REMS may strongly depend on parasympathetic cardiac modulation [31], which is severely reduced at low T_a_ in mice [39]. Central commands in REMS may also be reduced in frequency at low T_a_, as suggested by the reduced rate of occurrence of spontaneous SBP surges (Figure 7).

The present experiments evidenced a statistical tendency to a reduction in 24-h mean SBP value in TG compared with WT (*P* = 0.09, Figure 2 and Table S1), which we did not find in previous work [23]. This explains why in TG, blood pressure was lower than in WT in wakefulness and became the same in TG and WT during sleep in the present study (Figure 3), whereas it was the same in TG and WT during wakefulness and became higher in TG than in WT during sleep in our previous work [23], [28]. We are presently unable to provide a conclusive explanation of this difference. It is worth remarking that the present experimental protocol differed from that previously employed [23] in many respects, including a longer postoperative recovery, and that the genetic background of hybrid mice differs randomly between individuals, representing a potential source of variability in studies, such as ours, based on relatively small sample sizes. Nonetheless, the partial discrepancy between our previous [23], [28] and present work indicates that the effects of HCRT neuron loss on behaviour-related SBP fluctuations are more robust than, and partly distinct from, those on average 24-h SBP values and, thus, those on absolute values of SBP in each wake-sleep state. In fact, absolute values of SBP in each wake-sleep state may be viewed as behaviour-related SBP fluctuations superimposed on the 24-h average SBP. Accordingly, our present findings (Figure 3) confirm the blunted SBP decrease between wakefulness and the sleep states originally described in TG [23], demonstrating for the first time that it occurs irrespective of T_a_. Interestingly, reductions of sleep-related SBP fluctuations [24] and an impaired nocturnal dipping of diastolic blood pressure [40] have recently been found in patients with narcolepsy with cataplexy, who lack HCRT neurons, in the absence of significant changes in 24-h blood pressure values. The contribution of sympathetic nerve outflow to these findings was not measured in our present experiments and should be the subject of further study. Intracerebroventricular HCRT-1 administration at pharmacological doses increases renal sympathetic nerve activity in conscious rats [22]. However, in HCRT-deficient patients with narcolepsy-cataplexy, muscle sympathetic nerve activity is not reduced during rested wakefulness compared with controls and significantly increases during cataplexy [41].

In conclusion, our data demonstrate that at least in mice, HCRT neurons are not an essential part of the central neural mechanisms that coordinate thermoregulation with the control of wake-sleep behavior and the cardiovascular system. Our data also provide important new support of the view that HCRT neurons modulate the effects of sleep on the mean value of SBP, but not on SBP variability, indicating that these conclusions are not critically dependent on T_a_. Finally, our data demonstrate for the first time that in mice, the effects of sleep on cardiovascular control vary substantially, both quantitatively and qualitatively, even with modest changes in T_a_.

## Supporting Information

Table S1 **Daily profiles of wake-sleep behavior and cardiovascular variables: detailed results of the statistical analysis of variance.**
(DOC)

Table S2 **Effects of sleep and ambient temperature on systolic blood pressure and heart period: detailed results of the statistical analysis of variance.**
(DOC)

Table S3 **Total variability of systolic blood pressure and heart period and spontaneous baroreflex sensitivity: detailed results of the statistical analysis of variance.**
(DOC)

Table S4 **Cross-correlation functions between spontaneous fluctuations of heart period and systolic blood pressure: detailed results of the statistical analysis of variance.**
(DOC)

Table S5 **Cardiovascular features and frequency of occurrence of spontaneous blood pressure surges: detailed results of the statistical analysis of variance.**
(DOC)
