# Supplementary material for: Effects of Ambient Temperature on Sleep and Cardiovascular Regulation in Mice: The Role of Hypocretin/Orexin Neurons
Source: PLoS One. 2012 Oct 8;7(10):e47032. doi: 10.1371/journal.pone.0047032 (PMC3466227; doi:10.1371/journal.pone.0047032)
Supplement: Table S1 — Daily profiles of wake-sleep behavior and cardiovascular variables: detailed results of the statistical analysis of variance. (DOC) [file pone.0047032.s001.doc]

**Table S1. Daily profiles of wake-sleep behavior and cardiovascular variables: detailed results of the statistical analysis of variance**

|  | **Variable** | | | | |
| --- | --- | --- | --- | --- | --- |
| **Source** | **W** | **NREMS** | **REMS** | **SBP** | **HP** |
| group | 0.11 | 0.11 | 0.21 | 0.09 | 0.26 |
| T_a_ | **< 0.001** | **< 0.001** | **< 0.001** | **< 0.001** | **< 0.001** |
| LD | **< 0.001** | **< 0.001** | **< 0.001** | **< 0.001** | **< 0.001** |
| group x T_a_ | 0.35 | 0.37 | **0.04** | 0.56 | 0.11 |
| group x LD | 0.06 | 0.42 | **< 0.001** | 0.29 | 0.91 |
| T_a_ x LD | **< 0.001** | **< 0.001** | 0.30 | **0.049** | **< 0.001** |
| group x T_a_ x LD | 0.50 | 0.38 | 0.30 | 0.90 | 0.62 |

Data are significance (*P*) values of the analysis of variance (ANOVA) of the time spent in wakefulness (W), non-rapid-eye-movement sleep (NREMS) and rapid-eye-movement sleep (REMS), systolic blood pressure (SBP) and heart period (HP) as a function of ambient temperature (T_a_) and the light-dark period (LD) in orexin-ataxin3 transgenic mice (TG, n = 11) and wild-type controls (WT, n = 12). The between-subject factor was the mouse group (2 levels: TG and WT). The within-subject factors were ambient temperature (2 levels: 20 °C and 30 °C) and the light-dark period (2 levels: light and dark periods). The symbol x indicates interaction effects. *P* values < 0.05 are highlighted in red for clarity. Corresponding results are reported in Figure 2.
