# Supplementary material for: Effects of Ambient Temperature on Sleep and Cardiovascular Regulation in Mice: The Role of Hypocretin/Orexin Neurons
Source: PLoS One. 2012 Oct 8;7(10):e47032. doi: 10.1371/journal.pone.0047032 (PMC3466227; doi:10.1371/journal.pone.0047032)
Supplement: Table S3 — Total variability of systolic blood pressure and heart period and spontaneous baroreflex sensitivity: detailed results of the statistical analysis of variance. (DOC) [file pone.0047032.s003.doc]

**Table S3. Total variability of systolic blood pressure and heart period and spontaneous baroreflex sensitivity: detailed results of the statistical analysis of variance**

|  | **Variable** | | |
| --- | --- | --- | --- |
| **Source** | **SDSBP** | **SDHP** | **BRS** |
| group | 0.23 | 0.12 | 0.30 |
| T_a_ | 0.63 | **< 0.001** | **< 0.001** |
| state | **< 0.001** | **< 0.001** | **< 0.001** |
| group x T_a_ | 0.58 | 0.09 | 0.20 |
| group x state | 0.59 | 0.06 | 0.19 |
| T_a_ x state | **< 0.001** | **< 0.001** | **< 0.001** |
| group x T_a_ x state | 0.72 | 0.29 | 0.18 |

Data are significance (*P*) values of the analysis of variance (ANOVA) of the total variability of systolic blood pressure (SDSBP) and heart period (SDHP) and spontaneous baroreflex sensitivity (BRS) as a function of ambient temperature (T_a_) and the wake-sleep state in orexin-ataxin3 transgenic mice (TG, n = 11) and wild-type controls (WT, n = 12). The between-subject factor was the mouse group (2 levels: TG and WT). The within-subject factors were ambient temperature (2 levels: 20 °C and 30 °C) and the wake-sleep state (3 levels: wakefulness, non-rapid-eye-movement sleep, and rapid-eye-movement sleep). The symbol x indicates interaction effects. *P* values < 0.05 are highlighted in red for clarity. Corresponding results are reported in Figure 5.
