# Supplementary material for: Effects of Ambient Temperature on Sleep and Cardiovascular Regulation in Mice: The Role of Hypocretin/Orexin Neurons
Source: PLoS One. 2012 Oct 8;7(10):e47032. doi: 10.1371/journal.pone.0047032 (PMC3466227; doi:10.1371/journal.pone.0047032)
Supplement: Table S4 — Cross-correlation functions between spontaneous fluctuations of heart period and systolic blood pressure: detailed results of the statistical analysis of variance. (DOC) [file pone.0047032.s004.doc]

**Table S4. Cross-correlation functions between spontaneous fluctuations of heart period and systolic blood pressure: detailed results of the statistical analysis of variance**

|  | **Variable** | |
| --- | --- | --- |
| **Source** | **CCF peak** | **CCF trough** |
| group | **0.04** | 0.38 |
| T_a_ | **< 0.001** | **< 0.001** |
| state | **< 0.001** | 0.053 |
| group x T_a_ | 0.22 | 0.43 |
| group x state | 0.35 | 0.27 |
| T_a_ x state | **< 0.001** | **< 0.001** |
| group x T_a_ x state | 0.06 | 0.29 |

Data are significance (*P*) values of the analysis of variance (ANOVA) of the positive peak and the negative trough of cross-correlation functions (CCF) between spontaneous fluctuations of heart period and systolic blood pressure in orexin-ataxin3 transgenic mice (TG, n = 11) and wild-type controls (WT, n = 12). The between-subject factor was the mouse group (2 levels: TG and WT). The within-subject factors were ambient temperature (2 levels: 20 °C and 30 °C) and the wake-sleep state (3 levels: wakefulness, non-rapid-eye-movement sleep, and rapid-eye-movement sleep). The symbol x indicates interaction effects. *P* values < 0.05 are highlighted in red for clarity. Corresponding results are reported in Figure 6.
