# Supplementary material for: Effects of Ambient Temperature on Sleep and Cardiovascular Regulation in Mice: The Role of Hypocretin/Orexin Neurons
Source: PLoS One. 2012 Oct 8;7(10):e47032. doi: 10.1371/journal.pone.0047032 (PMC3466227; doi:10.1371/journal.pone.0047032)
Supplement: Table S5 — Cardiovascular features and frequency of occurrence of spontaneous blood pressure surges: detailed results of the statistical analysis of variance. (DOC) [file pone.0047032.s005.doc]

**Table S5. Cardiovascular features and frequency of occurrence of spontaneous blood pressure surges: detailed results of the statistical analysis of variance**

|  | **Variable** | | | |
| --- | --- | --- | --- | --- |
| **Source** | **ΔSBP peak** | **ΔHP trough** | **ΔHP**  **peak** | **Surge frequency** |
| group | 0.21 | 0.13 | **< 0.01** | 0.08 |
| T_a_ | **< 0.001** | **< 0.001** | **< 0.001** | **< 0.01** |
| state | **< 0.001** | **< 0.001** | **< 0.001** | **< 0.001** |
| group x T_a_ | 0.68 | 0.06 | **0.04** | 0.64 |
| group x state | 0.42 | 0.56 | 0.09 | 0.57 |
| T_a_ x state | **< 0.01** | **< 0.001** | **< 0.001** | 0.15 |
| group x T_a_ x state | 0.95 | 0.06 | **< 0.01** | 0.27 |

Data are significance (*P*) values of the analysis of variance (ANOVA) of the cardiovascular features and frequency of occurrence of spontaneous blood pressure surges as a function of ambient temperature (T_a_) and the wake-sleep state in orexin-ataxin3 transgenic mice (TG, n = 11) and wild-type controls (WT, n = 12). The analysis was performed on values of systolic blood pressure corresponding to the surge peak (ΔSBP peak), on the minimum value of heart period before the pressure surge peak (ΔHP trough), on the maximum value of heart period after the pressure surge peak (ΔHP peak), and on the frequency of occurrence of pressure surges. The between-subject factor was the mouse group (2 levels: TG and WT). The within-subject factors were ambient temperature (2 levels: 20 °C and 30 °C) and the wake-sleep state (3 levels: wakefulness, non-rapid-eye-movement sleep, and rapid-eye-movement sleep). The symbol x indicates interaction effects. *P* values < 0.05 are highlighted in red for clarity. Corresponding results are reported in Figure 7.
